# Robust Ground Target Detection by SAR and IR Sensor Fusion Using Adaboost-Based Feature Selection

**DOI:** 10.3390/s16071117

**Published:** 2016-07-19

**Authors:** Sungho Kim, Woo-Jin Song, So-Hyun Kim

**Affiliations:** 1 Department of Electronic Engineering, Yeungnam University, 280 Daehak-Ro, Gyeongsan, Gyeongbuk 38541, Korea; 2 Department of Electrical Engineering, Pohang University of Science and Technology, Pohang, Gyeongbuk 37673, Korea; wjsong@postech.ac.kr; 3 Agency for Defense Development, 111 Sunam-dong, Daejeon 34186, Korea; 153074@add.re.kr

**Keywords:** synthetic aperture radar, infrared, target detection, sensor fusion, machine learning, feature selection, OKTAL-SE

## Abstract

Long-range ground targets are difficult to detect in a noisy cluttered environment using either synthetic aperture radar (SAR) images or infrared (IR) images. SAR-based detectors can provide a high detection rate with a high false alarm rate to background scatter noise. IR-based approaches can detect hot targets but are affected strongly by the weather conditions. This paper proposes a novel target detection method by decision-level SAR and IR fusion using an Adaboost-based machine learning scheme to achieve a high detection rate and low false alarm rate. The proposed method consists of individual detection, registration, and fusion architecture. This paper presents a single framework of a SAR and IR target detection method using modified Boolean map visual theory (modBMVT) and feature-selection based fusion. Previous methods applied different algorithms to detect SAR and IR targets because of the different physical image characteristics. One method that is optimized for IR target detection produces unsuccessful results in SAR target detection. This study examined the image characteristics and proposed a unified SAR and IR target detection method by inserting a median local average filter (MLAF, pre-filter) and an asymmetric morphological closing filter (AMCF, post-filter) into the BMVT. The original BMVT was optimized to detect small infrared targets. The proposed modBMVT can remove the thermal and scatter noise by the MLAF and detect extended targets by attaching the AMCF after the BMVT. Heterogeneous SAR and IR images were registered automatically using the proposed RANdom SAmple Region Consensus (RANSARC)-based homography optimization after a brute-force correspondence search using the detected target centers and regions. The final targets were detected by feature-selection based sensor fusion using Adaboost. The proposed method showed good SAR and IR target detection performance through feature selection-based decision fusion on a synthetic database generated by OKTAL-SE.

## 1. Introduction

**Figure 1:**
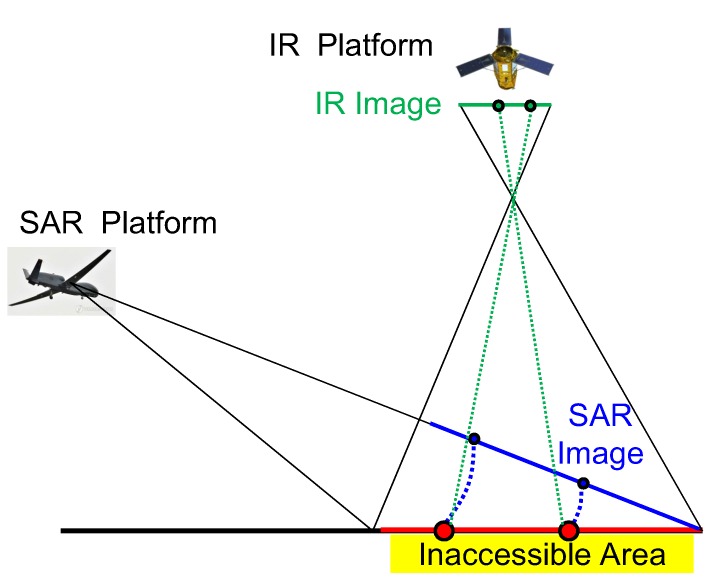
Operational concept of automatic target detection for inaccessible area surveillance.

Automatic target detection (ATD) is very important in military applications and there are challenging problems with ground surveillance [1]. Many studies have attempted to achieve high detection rates and low false alarm rates for well-known challenges, such as the target type, weather conditions, and background clutter [2,3,4]. On the other hand, the target detection problem is more difficult if the surveillance area cannot be accessed directly. Figure 1 shows a scenario of target detection in an inaccessible area using a multitude of sensor types, such as infrared (IR) and synthetic aperture radar (SAR), to enhance the detection performance. A SAR sensor mounted on an airplane can image an inaccessible area by the small depression angle and the IR sensor mounted on a satellite or unmanned aerial vehicle (UAV) can image the same region in a top-down view.

Targets in inaccessible areas can be detected by either the SAR sensor or IR sensor. SAR can measure the electromagnetic scattering property of targets under any weather and light conditions. This method is used frequently to detect distant targets because it provides strong radar cross section (RCS) values and shape information of non-stealth targets. On the other hand, it produces many false alarms due to speckle noise. In contrast, IR can provide thermal information and relatively informative shape information of the targets, despite being affected by the weather conditions, such as rain and fog [5,6]. IR sensors can provide target detection and cueing capabilities as well as the thermal signature of the detected targets, which can be a useful input for sensor fusion. In summary, the fusion of SAR and IR imagery enables a combination of complementary information, such as the thermal signatures in the IR case and RCS signatures in the SAR case. Both sensors have day and night capabilities while SAR sensor has weather-independency. Therefore, it is reasonable to use both SAR and IR sensors to detect targets stably in inaccessible areas by sensor fusion [7].

In general, the fusion of SAR and IR sensors for target detection can be described by the level at which the data is combined [8]. A pixel-level fusion scheme is used frequently in homogeneous sensors governed by the same underlying physical mechanisms, such and CCD and IR, which is an unsuitable fusion scheme in SAR and IR sensors because SAR and IR sensors measure completely different physical characteristics [9]. Feature-level fusion is performed on each sensor’s data individually, and these feature vectors are then combined before making decisions regarding target detection [10]. In contrast, individual detector decisions can be fused at the decision-level by fusing classifiers. According to this survey, most methods used either a feature-level or a decision-level fusion scheme for SAR and IR sensor. Few reports are available on SAR and IR fusion-based target detection due to military or security reasons. Dutkiewicz and Davenport proposed a structural element-based IR target detection method and K-distribution of RCS-based SAR target detection method for ship detection in 1996 [11]. They used two sensors for tie-point based alignment. In 1999 and 2000, Stephan and Childs introduced the SAR and IR fusion system briefly for ground surveillance [12,13]. Both SAR and IR used different target detection algorithms to localize potential targets and the final targets were detected at the decision level. Raghavan et al. used only SAR-based target detection for SAR-IR fusion-based recognition [14]. They used a constant false alarm rate (CFAR) detector to screen the targets in SAR images. Hero and Guillouet proposed a modified CFAR detector using the maximal invariants for SAR and IR detection [15]. Chen also used the well-known CFAR-based target detector to register SAR and IR images [16]. In this paper, a decision-level fusion scheme was adopted for SAR and IR-based target detection because independent processing can reduce the processing cost and less accurate sensor alignment is required. A feature-level fusion scheme requires relatively accurate sensor alignment and sliding window-like detection, which is unsuitable for heterogeneous SAR and IR sensors.

On the other hand, there are more studies related to single sensor-based ground target detection. In the case of the IR sensor-based target detection, a variety of filtering schemes, such as spatial filtering and spatio-temporal filtering have been proposed depending on the target motion and background clutter types [17]. In this study, stationary targets with a moving sensor platform were assumed. Therefore, spatial filter-based approaches can be feasible solutions. A background estimation and subtraction can provide satisfactory results if the target size is relatively small. A morphological nonlinear (top-hat) filter is a popular method [18,19]. Another linear filter is a modified mean subtraction filter (M-MSF), which is computationally simple and resistant to thermal noise [20]. The Max-Mean or Max-Median can be effective in target detection around a cloud edge [21]. Targets with different scales can be detected using a scale invariant approach in scale-space [22]. Recently, Qi et al. proposed Boolean map visual theory (BMVT)-based small target detection by fusing statistical orientation and intensity information [23].

In the case of SAR sensor-based target detection, a range of methods have been deployed, such as genetic programming [24], singular value decomposition [25], constant false alarm rate (CFAR) detector [26], and extended fractal feature-based detector [27]. The genetic programming-based detection method can achieve a low false alarm rate but it loses some information during statistical feature extraction [24]. The singular value decomposition approach uses high Eigen values that correspond to the SAR targets [25]. This method is theoretically good but it incurs huge computational cost. CFAR is a well-known target detector because it can detect potential targets effectively with low computational cost [26]. Different CFAR detectors can be used depending on the clutter models, such as the Weibull and K-distribution [26,28]. An order-statistics-based CFAR detector was proposed to reduce the effects of the clutter boundaries [29]. Because CFAR uses the signal intensity only, it produces too many target candidates [27]. Local signal distribution and phase-based SAR target detection methods have been proposed to overcome the limitations of intensity-based methods [30,31]. Recently, the max-margin based target detection method was proposed [32]. This method uses I-RELIEF feature weighting and change detection to make the detector robust to noise and unexpected clutter. The autocorrelation-based active contour method showed promising SAR target detection results [33].

**Figure 2:**
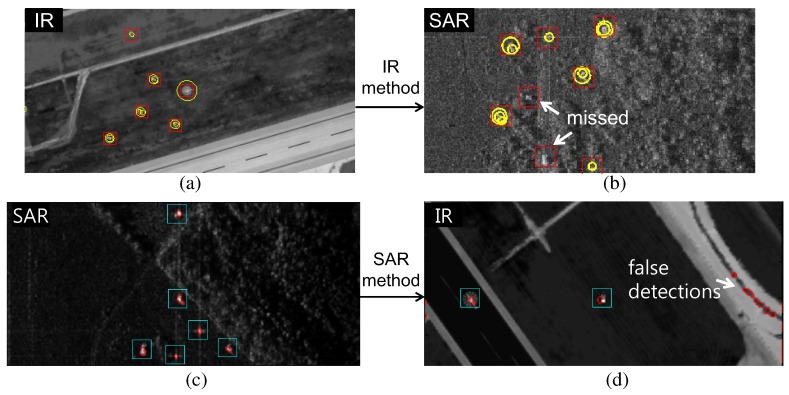
Problems of SAR and IR target detection by applying crossed methods: (**a**) IR target detection results using a Scale Invariant IR target detector [22]; (**b**) SAR target detection results using the same IR target detector; (**c**) SAR target detection results using an Active Contour-based SAR target detector [33]; (**d**) IR target detection results using the same SAR target detector. The Rectangles denotes the ground truths and the rounded edges denotes the detection results.

In general, sensor-dependent, specific target detection algorithms show good target detection performance. That is, the SAR sensor requires a proper scatter noise reduction method and the IR sensor requires proper modeling of the thermal targets. Sensor fusion using sensor dependent algorithms can show the best target detection performance. On the other hand, it is more efficient to use a single algorithm for SAR- and IR-fusion based target detection. A single algorithm can provide cost-effective hardware implementation for real-time applications using a system on a chip (SOC), field programmable gate array (FPGA), or general purpose graphics processing unit (GP-GPU). Reduced tuning parameters can allow faster system optimization in outdoor environments. Finally, a single algorithm is easy to manage for debugging and updating purposes. Although a unified algorithm is ideally suitable, actual applications have shown disappointing results, as illustrated in Figure 2. A Scale Invariant target detector shows good performance in IR small target detection, as shown in Figure 2a [22]. If the method is applied to a SAR image, it misses several important targets, as shown in Figure 2b. An Active Contour-based method shows excellent performance in SAR target detection Figure 2c [33]. If the method is applied to an IR target detection problem, it produces disappointing results, as shown in Figure 2d. Therefore, it is difficult to find a single method that is suitable for both SAR and IR target detection.

Based on these motivations, this paper proposes a single detection method to detect both SAR and IR targets simultaneously for a SAR and IR fusion study. In this study, a Boolean Map Visual Theory (BMVT)-based target detector was used as the baseline method [23]. Although the BMVT is effective for small IR target detection, it shows weak performance on extended IR and SAR targets. The original method was modified by inserting a median local average filter and an asymmetric morphological closing filter (called modBMVT) to handle the simultaneous SAR and IR target detection problem. Another contribution is the use of automatic SAR and IR image registration by RANdom SAmple Region Consensus (RANSARC) optimization on the detected target regions. The final contribution is the demonstration of target detection by Adaboost-based SAR and IR fusion. The proposed sensor fusion strategy should be assessed for target detection, even though deep learning algorithms are advanced techniques and have been provided to outperform in CCD-based pedestrian detection [34,35]. The deep learning algorithms require huge training images and are optimized for classification not detection or sensor fusion problems. Therefore, the proposed sensor fusion strategy should be assessed to improve the target detection performance.

The remainder of this paper is organized as follows. Section 2 introduces the background of BMVT theory and its limitations on the SAR/IR target detection problem. Section 3 explains the overall structure of the paper including the modBMVT-based SAR and IR target detection, RANSARC-based automatic SAR and IR registration, and sensor fusion-based final detection. Section 4 explains the preparation of the SAR and IR target database and an evaluation of the target detection performance of the proposed method. The paper is concluded in Section 5.

## 2. Background of BMVT Theory and Its Limitations

**Figure 3:**
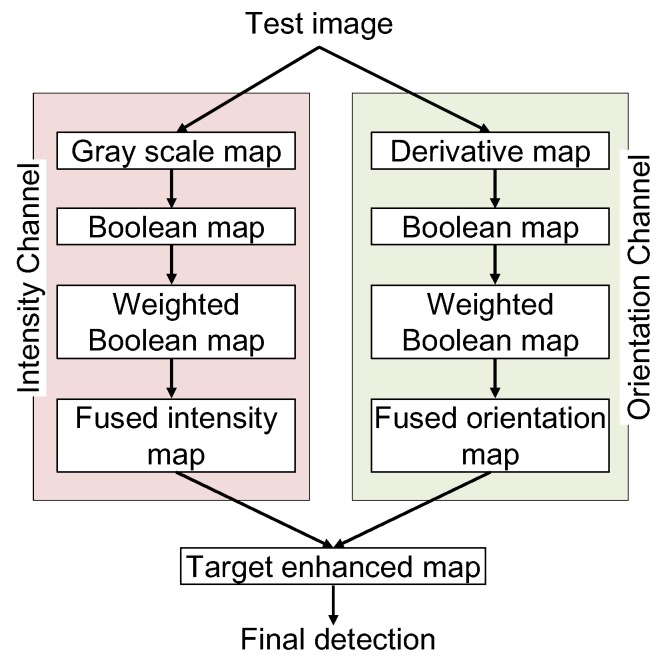
BMVT-based small IR target detection flow [23].

Many small IR target detectors are available, as mentioned above. In particular, the Boolean Map Visual Theory (BMVT)-based detector shows outperforming results for small IR target detection in a cluttered environment [23]. This is based on the Boolean Map theory of human visual attention [36]. Human visual systems extract each feature (Boolean or binary map) and generate new features by associating each feature using Boolean AND/OR operations to find the attention points. Qi et al. applied Boolean Map Visual Theory to find small infrared targets that exhibit the highest attention by incorporating intensity channels and orientation channels, as shown in Figure 3. The BMVT-based detection method separates a test image into an intensity channel and orientation channel.

In an intensity channel, the original format of an input image (12 bits or 14 bits) is changed to a 8 bit gray scale image ($F_{i}\left(x,y\right)$) at the pixel position, (x,y). Boolean maps ($B_{ij}\left(x,y\right)$) of the gray image are generated by thresholding with fixed intervals ($T_{j}$), as expressed in Equation (1).

(1) $$\begin{array}{c} B_{ij}=\left\{\begin{array}{cc} & \;1\;\;\text{if}\;F_{i}\left(x,y\right)\geq T_{j}\\ & \;0\;\;\text{else} \end{array}\right. \end{array}$$

An intensity interval is normally selected as 4 considering the computational complexity. In this case, the total number of Boolean maps can be 64 (256/4). A weighted Boolean map is produced based on the statistical distribution of the Boolean map. Large weights are applied to the Boolean map if the number of foreground pixels (labeled as 1) is small compared to the total number of image pixels, as expressed in Equation (2).

(2) $$\begin{array}{c} \omega =\frac{N_{tot}}{N_{fg}} \end{array}$$

This is the same as the attention property of the human visual system. The human visual system gives high weight to distinctive points. An intensity fused Boolean map ($B_{i}^{F}$) is obtained by summing the weighted Boolean maps, as expressed in Equation (3), where $\omega_{ij}$ denotes the weight of $B_{ij}$. In the case of the intensity channel, *i* is the same as 1 and *j* equals the total number of intensity levels.

(3) $$\begin{array}{c} B_{i}^{F}=\sum_{j}\omega_{ij}B_{ij} \end{array}$$

**Figure 4:**
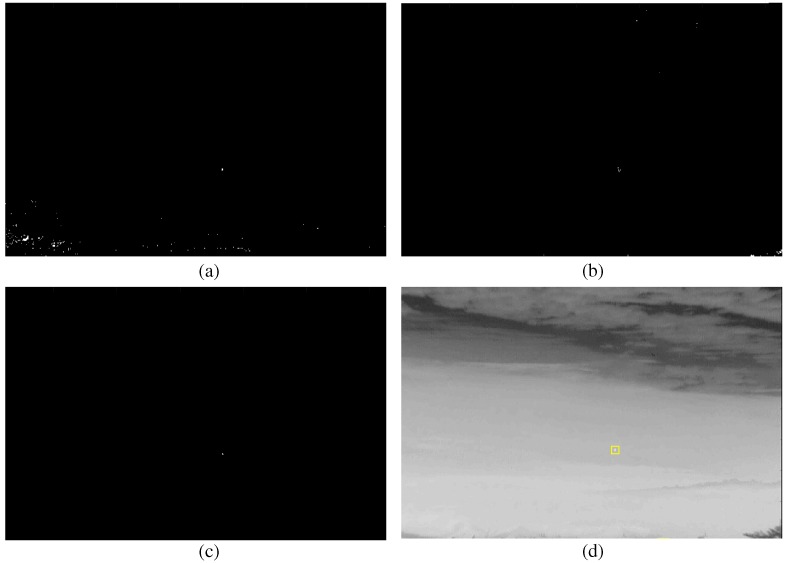
Successful small infrared target detection results: (**a**) fused intensity map; (**b**) fused orientation map; (**c**) enhanced map; (**d**) final detection.

**Figure 5:**
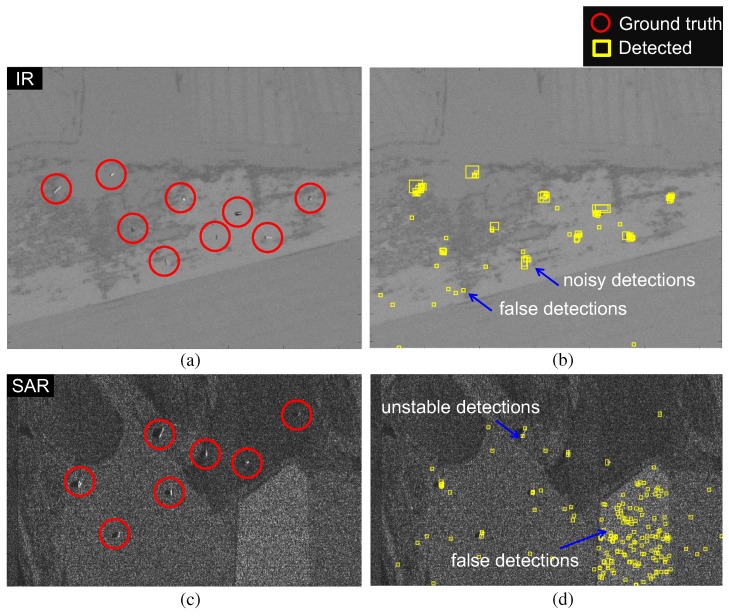
Problems with the original BMVT-based SAR/IR target detection: (**a**) IR test image; (**b**) BMVT-based IR target detection; (**c**) SAR test image; (**d**) BMVT-based SAR target detection.

In an orientation channel, directional derivative feature maps ($F_{i}^{O}\left(x,y\right)$) are calculated using the second order directional derivative (Laplacian) kernels to enhance blob-like or convex targets. The remaining steps of Boolean map extraction and fused Boolean map generation are the same as written in Equations (1)–(3) except that the index of *i* depends on the number of orientations. Because the number of fused maps of the orientation channel can be 4 or 8, an additional fused Boolean map ($B_{c}^{F}$) should be generated using Equation (4).

(4) $$\begin{array}{c} B_{c}^{F}=\frac{1}{|\Omega_{c}|}\sum_{i\in \Omega_{c}}B_{i}^{F} \end{array}$$

where $\Omega_{c}$ denotes the index set of orientations. A larger number of orientation channels can enhance the detection accuracy but with the increased computational cost. The target enhanced map ($I_{E}$) can be generated by multiplying the fused maps of the intensity and orientation channels, as expressed in Equation (5).

(5) $$\begin{array}{c} I_{E}=\prod_{c\in C}B_{c}^{F} \end{array}$$

where *C* represents the index set of the fused intensity and orientation channels. Small IR targets are enhanced by considering both the intensity and orientation. Therefore, the target candidates of the bright circular spots can be detected easily by applying binarization to $I_{E}$. Figure 4d gives an example of successful small IR target detection for a normal aerial target. The enhanced map (Figure 4c) was obtained by multiplying the fused intensity map (Figure 4a) and orientation map (Figure 4b). The BMVT works quite well if the target size is very small (approximately 3–50 pixels) and the background is homogeneous. On the other hand, it shows disappointing detection performance if the method is applied to detect extended targets (approximately 50–200 pixels), as shown in Figure 5. Figure 5a,c shows IR and SAR test images including the ground truth regions. The test images were generated using the SAR/IR image generator, OKTAL-SE, which will be explained in the experimental section [37]. Figure 5b,d shows the detection results using the BMVT method. The detection results are quite noisy around the target regions. In addition, the BMVT is quite sensitive to thermal noise in an IR image and scattering noise in a SAR image. Such phenomena originate from the point-like bright spot detection capability of BMVT-based detection. The intensity channel emphasizes the bright regions and the orientation channel emphasizes the circular or convex regions.

## 3. Proposed Fusion-Based Extended SAR/IR Target Detection Using modBMVT

**Figure 6:**
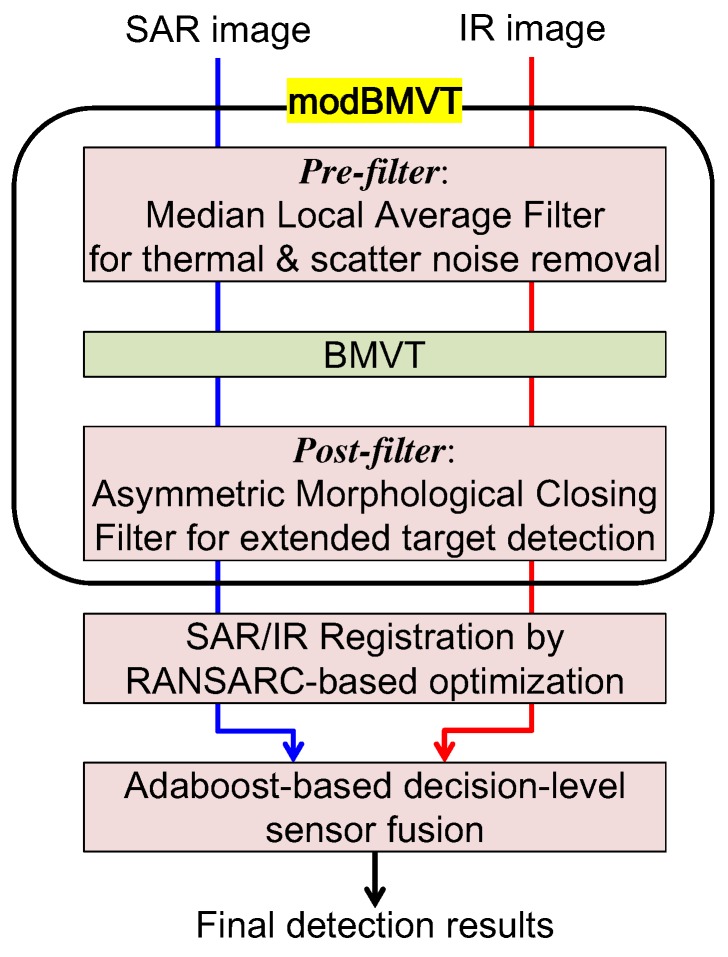
Proposed decision level fusion-based extended SAR/IR target detection using modBVMT and RASARC-based registration.

The proposed fusion-based target detection consists of modified BMVT-based basic detection block, automatic SAR/IR registration block, and logical fusion block, as shown in Figure 6. Details of each block will be handled in the following sub-blocks.

### 3.1. Proposed Modified BMVT-Based Target Detection: modBMVT

As mentioned in the previous section, the original BMVT-based target detection has two problems if the method is applied to extended SAR/IR target detection. The first is that it generates many false detections due to thermal noise in the IR images and scattering noise in the SAR images. The second is that it produces partitioned partial detection to the extended ground targets.

**Figure 7:**
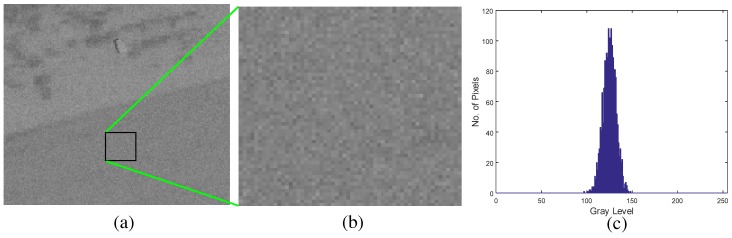
Thermal noise analysis in an infrared image: (**a**) test sample image; (**b**) enlarged image of background region; (**c**) noise distribution.

**Figure 8:**
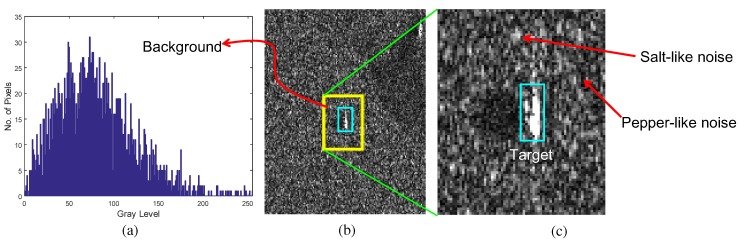
Proposed decision level fusion-based extended SAR/IR target detection using modBVMT and RANSARC-based registration: (**a**) distribution of background region, (**b**) target and background region, and (**c**) salt and pepper noise.

The first problem can be solved by inserting a hybrid noise reduction filter in front of the BMVT detector. If the thermal noise is analyzed, the noise distribution is similar to the Gaussian distribution, as shown in Figure 7. The target signal or background intensity can be estimated optimally by applying the expectation to the Gaussian random variables. The unbiased optimal estimator is the sample average or linear mean filter [38]. In the case of an SAR image, the noise characteristics are quite different to the IR case. Owing to the electro-magnetic scattering phenomena during SAR imaging, the noise distribution can be modeled as Weibull, log-normal, or Fisher depending on the conditions, as shown in Figure 8 [39]. If the background pixels are enlarged, as shown in Figure 8c, the noise pixels show salt and pepper-like intensities. This noise can lead to false detection in the BMVT-based detector but can be removed using an order-statistics filter, such as a median filter [40]. In this paper, the original SAR intensity is used to preserve the target signature instead of a logarithmic scale where the amplitude distribution can be approximately Gaussian.

The main question is, how can both thermal noise and scattering noise be removed? This paper proposes a novel median local average filter (MLAF), which is a hybrid version of the mean and median filters, to remove heterogeneous noise from IR and SAR images, as expressed in Equation (6).

(6) $$\begin{array}{c} \hat{F}\left(x,y\right)=\frac{1}{d}\sum_{\left(s,t\right)\in S_{xy}}G_{m}\left(s,t\right) \end{array}$$

**Figure 9:**
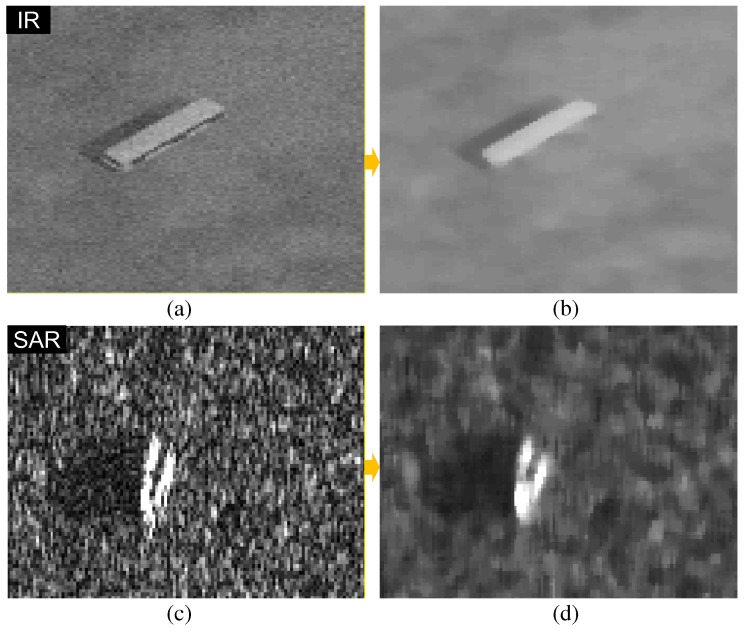
Proposed MLAF-based thermal and scattering noise reduction results: (**a**) noisy IR image; (**b**) MLAF-based noise reduction; (**c**) noisy SAR image; (**d**) MLAF-based noise reduction.

Pixels in the filtering region (size n×n) of an input image (F(x,y)) are sorted in descending order. The *d* intensity values around a median intensity of G(s,t) in the neighborhood $S_{xy}$ are averaged. Let $G_{m}\left(s,t\right)$ represent the selected *d* pixels. A median filter can remove the salt-pepper noise and the local average can remove the Gaussian noise effectively. Therefore, the proposed MLAF can handle the heterogeneous noise. Normally, the neighboring window size is 5×5 and d=5. The selection of parameter *d* is important. The salt noise shows a high intensity rank and the pepper noise shows low intensity rank. The size *d* is calculated by d=(n×n)×percentagearoundmedian(δ). The signal-to-clutter ratio (SCR) vs. percentage around the median curve is plotted in Figure 22a. The best SCR performance is observed around 10%∼20% of the median. If *δ* is 20% and *n* is 5, d=(5×5)×20%=5. Therefore, the optimal d is selected experimentally. Figure 9 shows the effects of the proposed MLAF noise reduction filter on both IR and SAR images. Note that the MLAF can remove both thermal noise and scattering noise using a single framework and parameters while preserving the target shapes. The roles of MLAF can be analyzed in the experimental section in terms of the signal-to-clutter ratio (SCR).

**Figure 10:**
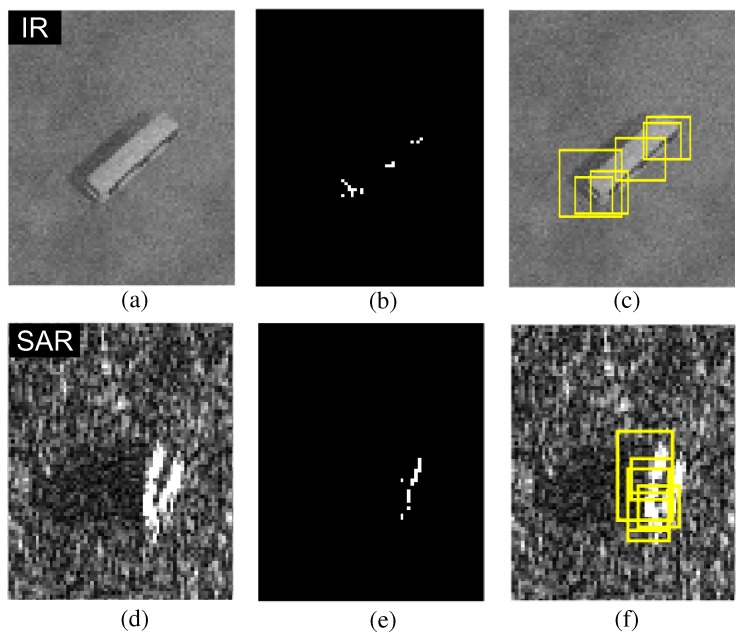
Problems of the original BMVT-based extended target detection: (**a**) extended IR target image; (**b**) IR target extracted map by applying a threshold to the enhanced map; (**c**) 8-nearest neighbor-based final IR target detection results; (**d**) extended SAR target image; (**e**) SAR target extracted map; (**f**) final SAR target detection results.

The second problem can be solved by attaching a morphological closing filter after the BMVT detector. The original BMVT-based target detector generates partitioned or fractional detection results, as shown in Figure 10 for both extended IR and SAR targets. The BMVT can detect only small point-like targets, which leads to fractional detection, as shown in Figure 10b,d. The final detections were obtained by applying 8-nearest neighbor-based clustering.

The fractional detection problem can be alleviated in several aspects through the procedures of the BMVT. The second order directional derivative kernel can be applied to a scale-space image to handle the extended targets. On the other hand, this approach cannot provide satisfactory detection results. In this study, an asymmetric morphological closing filter (AMCF) was applied to the binarized enhanced map ($I_{B}$) with a structural element (SE), as expressed in Equation (7).

(7) $$\begin{array}{c} I_{AMCF}\left(x,y\right)=\left(I_{B}\left(x,y\right)⊕SE\left(k,k\right)\right)⊙SE\left(l,l\right) \end{array}$$

**Figure 11:**
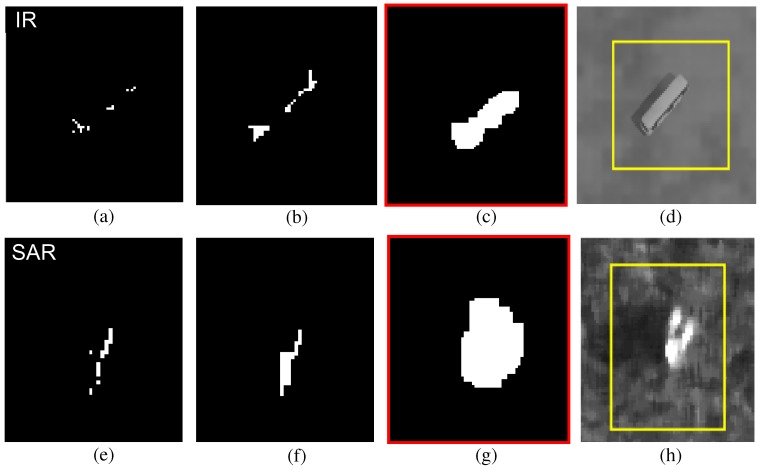
Asymmetric morphological closing filter (AMCF)-based IR/SAR target detection results: (**a**) initial IR target detection in BMVT; (**b**) conventional symmetric morphological closing filter (IR, k=18,l=18); (**c**) proposed asymmetric morphological closing filter (IR, k=18,l=10); (**d**) detected IR target; (**e**) initial SAR target detection in BMVT; (**f**) conventional symmetric morphological closing filter (SAR, k=18,l=18); (**g**) proposed asymmetric morphological closing filter (SAR, k=18,l=10; (**h**) detected SAR target.

The binary enhanced map is dilated (⊕) with k×k structural square elements. The dilated map is eroded (⊙) by the second structural square elements with a size of l×l. Normally, *k* is larger than *l* because the dilation process requires a large size of structural elements to merge the scattered pixels and the erosion process requires relatively small structural elements to make the eroded target convex. This process is called the asymmetric morphological closing filter (AMCF) due to the different size of its structural elements. In these experiments, the optimally tuned parameter values were set to k=18 and l=10. In general, *k* controls the gap size to link the detected pixels. Therefore, *k* is set as the maximum gap size after BMVT-based detection. On the other hand, *l* controls the erosion size. The *l* value can be selected depending on the final target size. If a target width after AMCF is 8 for a 1 pixel width target, then *l* can be calculated by l=k−8. Figure 11 shows the improved target detection results by applying the proposed AMCF to the results of the BMVT in the IR and SAR images. The AMCF was applied after the BMVT to connect the fractional target pixels. Figure 11b presents a conventional closing filter applied to the IR image (k=18,l=18) and Figure 11c shows the proposed AMCF result applied to the IR image (k=18,l=10). If a conventional symmetric morphological closing filter is applied, the results are thinned or partly disconnected, as shown in Figure 11b,f. The same AMCF works for SAR target detection, as shown in Figure 11g,h. If Figure 10 and Figure 11 are compared, the proposed AMCF is effective in extended IR and SAR target detection. Note that the same pre-filter (MLAF) and post-filter (AMCF) were used in the IR and SAR images to detect the targets stably.

### 3.2. RANSARC-Based SAR/IR Registration

**Figure 12:**
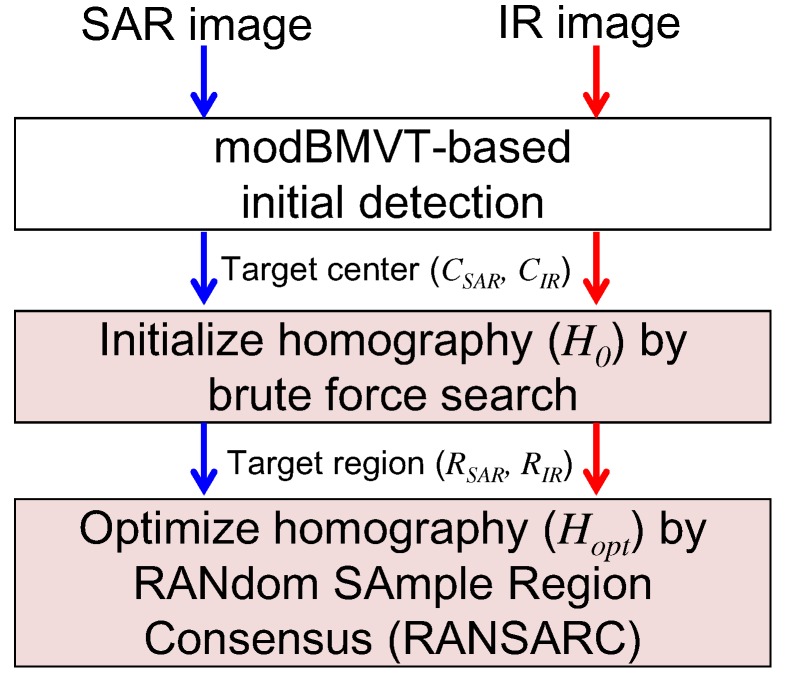
Proposed detection-based SAR/IR registration using RANSARC optimization.

Aligning or registering the SAR and IR images is important for detecting the targets by sensor fusion. A direct pixel matching-based registration approach is impractical because the SAR and IR images have different physical characteristics and imaging geometries (SAR: slanted viewing direction; IR: top-down viewing direction). In this paper, a novel SAR and IR registration scheme was applied, as shown in Figure 12. The scheme consists of estimating the initial image homography ($H_{0}$) and optimizing image homography ($H_{opt}$). The key idea is to utilize the initial SAR and IR target detection results to find the correspondence. The modBMVT produces both target centers ($C_{SAR},C_{IR}$) and target regions ($R_{SAR},R_{IR}$) from each sensor image.

**Figure 13:**
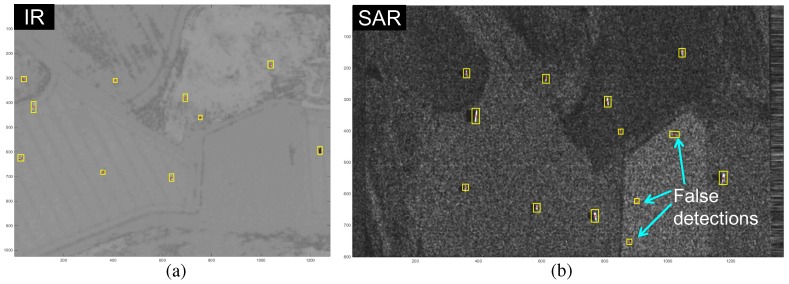
modBMVT-based initial target detection including the target centers: (**a**) IR target detection (BG2-T10-IR); (**b**) SAR target detection (BG2-T10-SAR). The code, BG2-T10-IR/SAR, denotes (background type-number of targets-sensor type).

As shown in Figure 13, the initial detections are overlaid on the input IR and SAR images including the target centers (red crosses). Two different images can be registered using image homography [41]. The fundamental problem is that the initial matching pairs between the IR and SAR image pixels to estimate image homography are not known (3×3matrix,8unknowns). If the same image sensors are used, local invariant features, such as the SIFT, can provide stable image matches in image registration [42]. The IR camera can sense thermal radiation emitted from the targets and background. The SAR device can sense the electro-magnetic scattering reflected from the targets and backgrounds. The same targets show completely different intensity and shape characteristics, as shown in Figure 13.

This paper proposes a brute-force search and consensus matching method to find the initial image homography ($H_{0}$) using the detected target centers. Assume that *p* IR targets and *q* SAR targets are detected, including true targets and false detections. The first step is to prepare all possible 4-point matching combinations ($N_{tot}$), as expressed in Equation (8).

(8) $$\begin{array}{c} N_{tot}{=}_{p}C_{4}^{IR}\times_{q}C_{4}^{SAR} \end{array}$$

**Figure 14:**
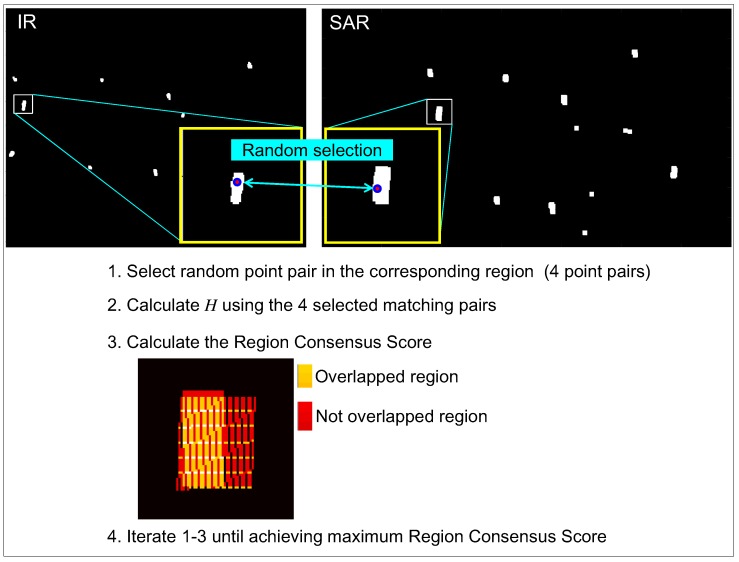
Proposed homography optimization by RANdom SAmple Region Consensus (RANSARC).

The next step is to calculate the image homogrphy for each matching combination [41]. The final step is to select the best homography that maximizes the matching consensus of the remaining matching points, such as the RANSAC method [43]. The estimated initial homography ($H_{0}$) is inaccurate because the target center points detected are affected by the image noise and detecting environments. This paper proposes a novel homography optimization method by applying RANdom SAmple Region Consensus (RANSARC) optimization to the detected SAR/IR regions, as shown in Figure 14. Given the SAR and IR matching information and detected regions ($R_{SAR},R_{IR}$), 4 random matched points were selected from the corresponding matched regions. The next step was to calculate the hypothesized homography and region consensus score using Equation (9), where $R_{SAR}^{T}$ denotes the transformed SAR region using the hypothesized homography.

(9) $$\begin{array}{c} Score\left(R_{IR},R_{SAR}^{T}\right)=\frac{R_{IR}\cap R_{SAR}^{T}}{R_{IR}\cup R_{SAR}^{T}} \end{array}$$

**Figure 15:**
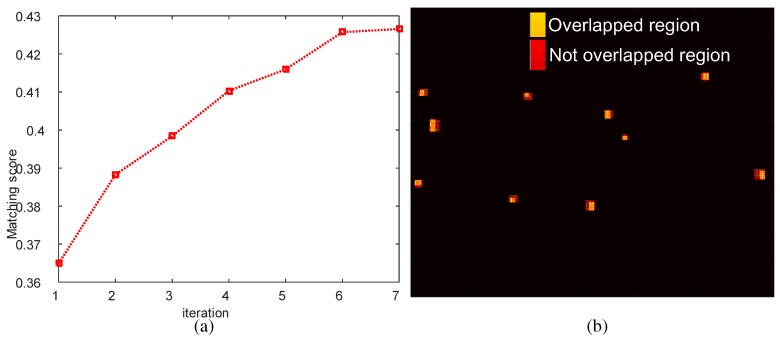
RANSARC-based homography optimization results: (**a**) matching score graph; (**b**) region overlap image using the optimized homography ($H_{opt}$).

**Figure 16:**
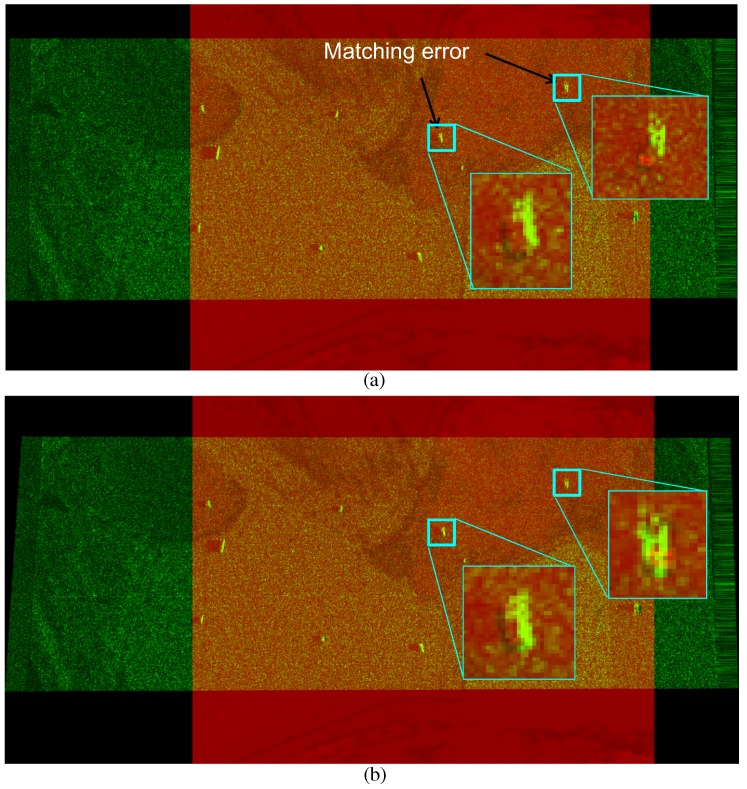
Comparisons of the image registration using (**a**) initial homography estimated by a brute-force search from the target points; (**b**) additionally optimized homography by the proposed RANSARC.

This process continues until the maximum score is achieved. Figure 15 presents the region score optimization results and final region overlap image using the optimized homography. The yellow dots represent the region overlap between the IR and SAR image. Figure 16 compares the image registrations conducted by the initial homography ($H_{0}$) and optimized homography ($H_{opt}$) estimated by RANSARC. The SAR image is represented as the green channel and the IR image is represented as the red channel. Note that the two targets indicated by the arrows were misaligned using the initial homography, as shown in Figure 16a. The proposed RANSARC can reduce these errors, as shown in Figure 16b.

### 3.3. Target Detection by Adaboost-Based SAR/IR Fusion

**Figure 17:**
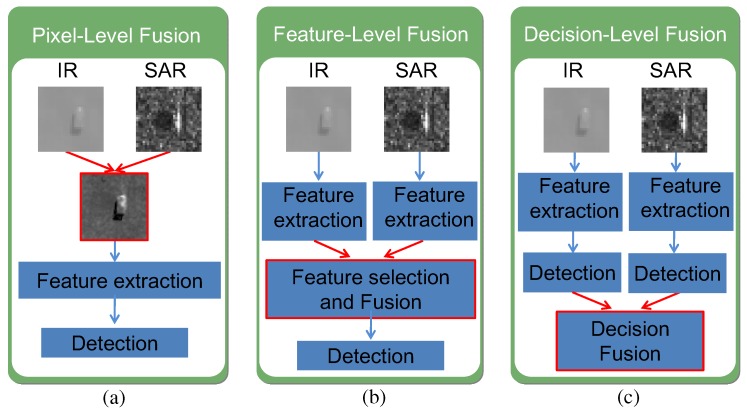
SAR and IR sensor fusion-based target detection schemes: (**a**) pixel-level fusion; (**b**) feature-level fusion; (**c**) decision-level fusion.

The final targets can be detected by SAR and IR detection fusion given the SAR and IR detection and registration information. The detection of targets from multisensor data can be described at the level at which the data are combined [8]. According to the sensor fusion strategy, there are three kinds of fusion schemes in SAR and IR fusion-based detection, as shown in Figure 17 [44,45]. The pixel-level fusion scheme is used frequently in homogeneous sensors governed by the same underlying physical mechanisms, such and CCD and IR, or in visualization for human understanding [9]. In pixel-level fusion, the data is combined, the features are then extracted. In contrast, in feature-level fusion, feature extraction is performed individually on the data of each sensor, and these feature vectors are then combined. The concatenated feature vector of SAR and IR is used in the classifiers [10]. Individual detector decisions can be fused at the decision-level by a logical AND/OR operation [46], Dempster-Shafer method [47], or Bayesian method [48]. Target detection by either feature-level or decision-level sensor fusion is a feasible approach for heterogeneous sensors, such as SAR and IR.

This paper adopted the third fusion scheme, decision-level fusion, for target detection. Each sensor detects the candidate targets using the modBMVT method. The attribute features of the SAR and IR target, such as the filtered intensity and target area, are extracted using individual detection information generated by SAR and IR sensors. The ensemble classifiers learned by the Adaboost algorithm can decide the final detection. If $I_{k}^{SAR}$ denotes the maximum of asymmetric morphological closing filter at the $k^{th}$ region and $a_{k}^{SAR}$ denotes the area of a segmented target region, the total SAR/IR target feature of the $k^{th}$ ROI is defined as Equation (10). If only one sensor generates an ROI, the same ROI is used by the other sensor for feature extraction.

(10) $$\begin{array}{c} f_{k}={\left[I_{k}^{SAR}\;a_{k}^{SAR}\;I_{k}^{IR}\;a_{k}^{IR}\right]}^{T} \end{array}$$

The study investigated two kinds of feature selection methods using the Laplacian support vector machine (LapSVM) and Adaboost [49,50]. SVM is a well-known classifier that can provide feature-vector-based strong classification performance in a range of applications. This classifier uses the whole feature vectors (f) as support vectors in decision boundary learning using a kernel recipe. In contrast, Adaboost uses weak classifiers ($h_{i}$), such as thresholding, for each feature element. The combined weak classifier can be a strong classifier by learning or feature selection ($\alpha_{i}$), as expressed Equation (11). Details of fusion-based target detection results will be handled in the experimental section.

(11) $$\begin{array}{c} H_{strong}\left(f\right)=sign\left(\sum_{i}\alpha_{i}h_{i}\left(f\right)\right) \end{array}$$

## 4. Experimental Results

### 4.1. SAR/IR Database

In SAR and IR fusion-based target detection and recognition, the most difficult part is how to prepare the SAR and IR database (DB) for the same target and background environments to validate a range of detection algorithms. In this paper, the basic assumption is that only standing or non-moving vehicles are considered to prevent the misplacement of moving targets during SAR and IR image acquisition. Although IR cameras are usually static sensors, i.e., they acquire one image at one point in time, SAR sensors are by their very nature kinematic devices, which need to integrate high-frequent measurements over time to produce an image. This leads to the misplacement of moving objects in the resulting images.

**Figure 18:**
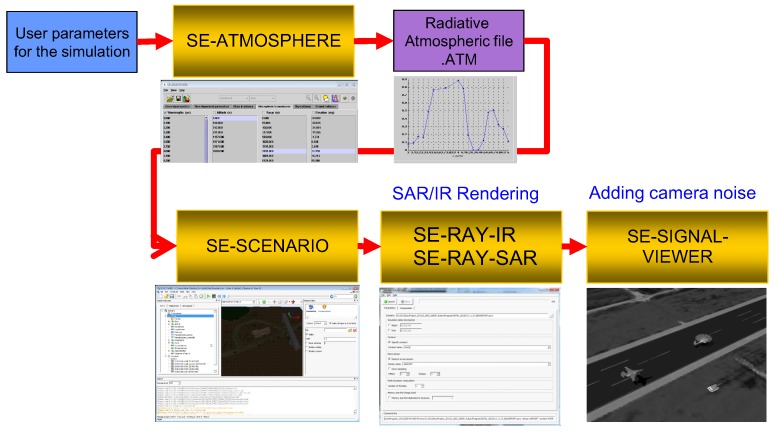
OKTAL-SE-based SAR and IR image generation flow.

**Figure 19:**
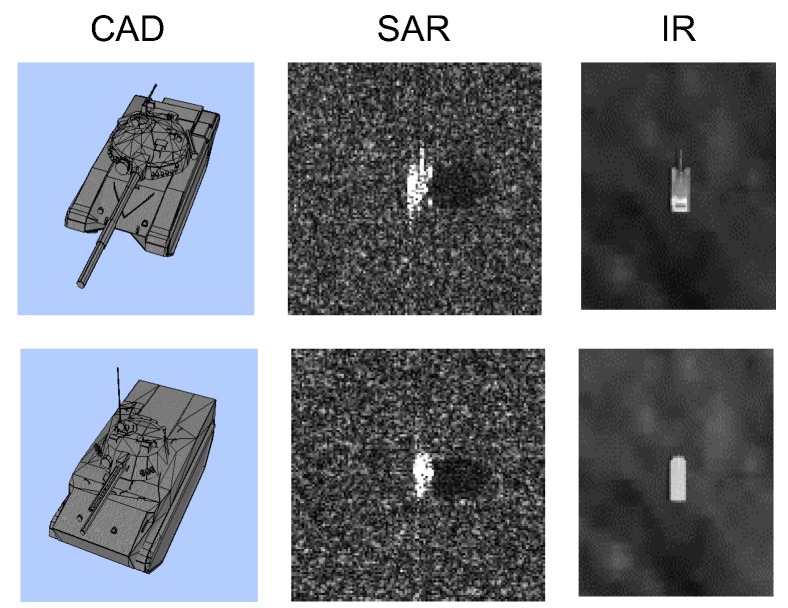
Examples of SAR and IR target generation using OKTAL-SE.

According this survey, there is no public SAR/IR database due to security reasons. Four kinds of DB preparation methods can be considered. The first database acquisition strategy is to use a real IR camera and SAR sensor mounted on an airplane. This is the most accurate and useful method but it incurs the highest cost due to the expensive sensors and acquisition platform. The second strategy is to use satellites, such as TerraSAR-X for SAR and KOMSAT-3A for IR. On the other hand, identifying the various military target images for both SAR and IR sensors is also very difficult and expensive. The third method is to develop a SAR and IR simulator for DB generation, which is out of the current research scope and will require a long time for development. The final strategy is to purchase commercially available software that can synthesize both SAR and IR images for the same scenario. Several simulators that can work on a specific spectrum are available: DIRSIG and SensorVision for IR and Xpatch for radar. The only simulator that can generate both SAR and IR images is OKTAL-SE, which is a proven synthesizing tool [37,51]. As shown in Figure 18, the user parameter and atmospheric file are inserted into the SE-SCENARIO program, which can manipulate the SAR/IR sensor platform and locate the targets in a specific background. SAR and IR images are generated simultaneously for the same scenario using SE-RAY-IR and SE-RAY-SAR software. The synthesized raw data can be modified further by reflecting the sensor noise in the SE-SIGNAL-VIEWER module. Figure 19 provides partial examples of a SAR and IR image generation for the T72 and BMP3 targets. The generated SAR image shows very strong scattering noise and the IR image shows a bright intensity around the engine location.

**Figure 20:**
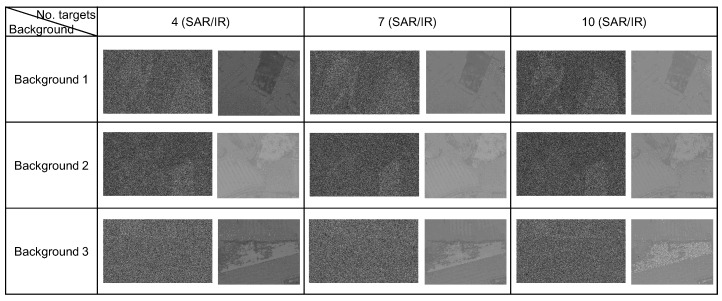
Synthesized SAR and IR database generated by OKTAL-SE.

The following parameters were used to make a DB for target detection purposes. The sensor to target distance was approximately 4.5 km with different depression angles: $15^{\circ }$ for SAR and $70^{\circ }$ for IR. The targets used in DB were BMP3, T72, AMX10, TMM, Radar camo, VAB OBS, Audi, Car supply, Bus, and Backstairs car. Figure 20 summarizes the prepared SAR and IR DB by varying the background types and number of targets.

### 4.2. Individual SAR/IR Target Detection by BMVT vs. Proposed modBMVT

**Figure 21:**
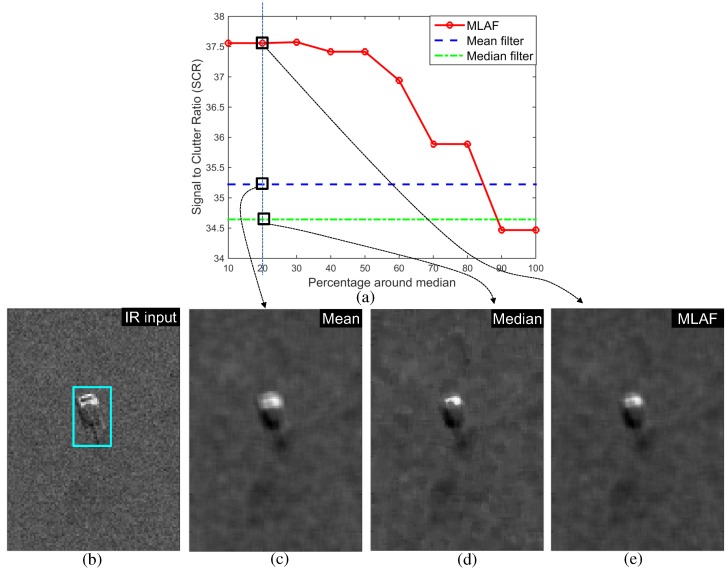
IR noise removal comparison: (**a**) signal-to-clutter ratio by varying the percentage around the median value in MLAF; (**b**) IR test image; (**c**) mean filter result (5×5); (**d**) median filter result; (**e**) proposed MLAF result at 20% around the median.

**Figure 22:**
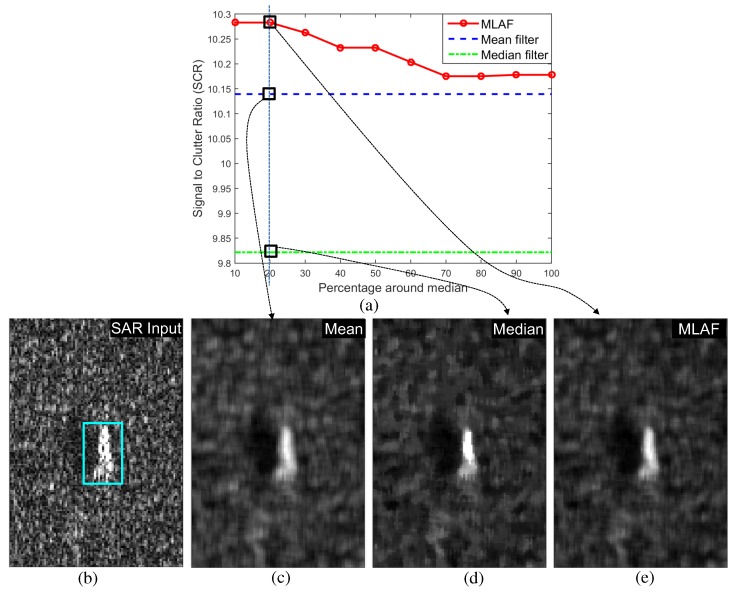
SAR noise removal comparison: (**a**) signal-to-clutter ratio by varying the percentage around the median value in MLAF; (**b**) IR test image; (**c**) mean filter result (5×5); (**d**) median filter result; (**e**) proposed MLAF result at 20% around the median.

This paper proposes a modified BMVT-based detection method by inserting the pre-filter and post-filter. The pre-filter, called the Median Local Average Filter (MLAF), can remove both thermal and scattering noise in an IR and SAR image. The noise removal capability was compared quantitatively in terms of the signal-to-clutter ratio (SCR) defined in Equation (12) [20], which is used frequently in the target detection problem. $\mu_{\hat{F}}^{T}$ denotes the mean target intensity and $\sigma_{\hat{F}}^{BG}$ denotes the standard deviation of the background region. If the SCR is increased after applying some spatial filter, the spatial filter is considered to be effective in noise removal. Figure 21 and Figure 22 show the quantitative evaluation results of the noise removal filter and related images for IR and SAR test images. As a baseline filtering method, the mean and median filters were used. Figure 21a was obtained by varying the percentage around the median in the MLAF with the same kernel size (5×5). The proposed method showed higher SCR values if the pixels with approximately 10%–30% of the median intensity are used in filtering. Figure 21b shows the test IR target and background image. Figure 21c–e represent the noise removal results using the mean, median and proposed MLAF (20%). The mean filter produced a blurred target and median filter distorted the target. On the other hand, the proposed MLAF shows a compromised result with a higher SCR value. This is the same as the scatter noise in the SAR image, as shown in Figure 22. Note that the proposed MLAF can provide a relatively clear target region in both the IR and SAR noisy images with increased SCR values compared to the conventional mean and median filter.

(12) $$\begin{array}{c} SCR=\frac{\mu_{\hat{F}}^{T}}{\sigma_{\hat{F}}^{BG}} \end{array}$$

**Figure 23:**
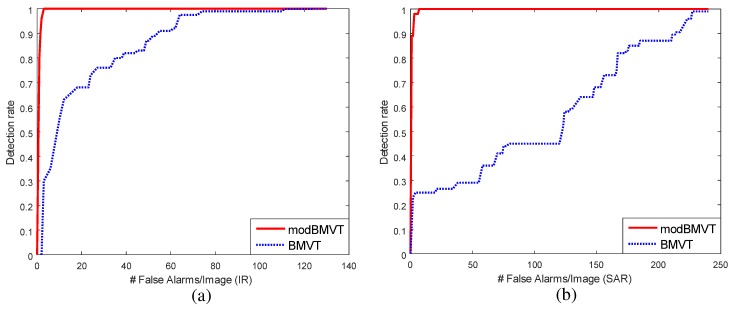
Performance evaluation of the individual target detection of the proposed modBMVT compared to the original BMVT: (**a**) ROC comparison for the IR test image of BG1-T10-IR; (**b**) ROC comparison for the SAR test image of BG1-T10-SAR.

The post-filter, which is called the asymmetric morphological closing filter (AMCF), is used to reduce the partitioned detection pixels in the extended SAR and IR detection. The proposed modBMVT-based target detection method is made by attaching a pre-filter (MLAF) and post-filter (AMCF) to the conventional BMVT method. Figure 23 summarizes the performance comparison in terms of the receiver operating characteristic (ROC) curves for both IR and SAR target images (background ID-target number-IR/SAR:BG1-T10-IR/SAR). The proposed modBMVT can provide upgraded ROC performance compared to the BMVT in both IR and SAR test images. Figure 5 shows the target detection results using a conventional BMVT-based method and Figure 13 shows the target detection results using the proposed modBMVT method with the specific threshold to meet the detection rates. Note that the proposed method can find the true target regions reasonably well with a lower number of false detections.

### 4.3. Evaluation of RANSARC-Based SAR/IR Registration

**Figure 24:**
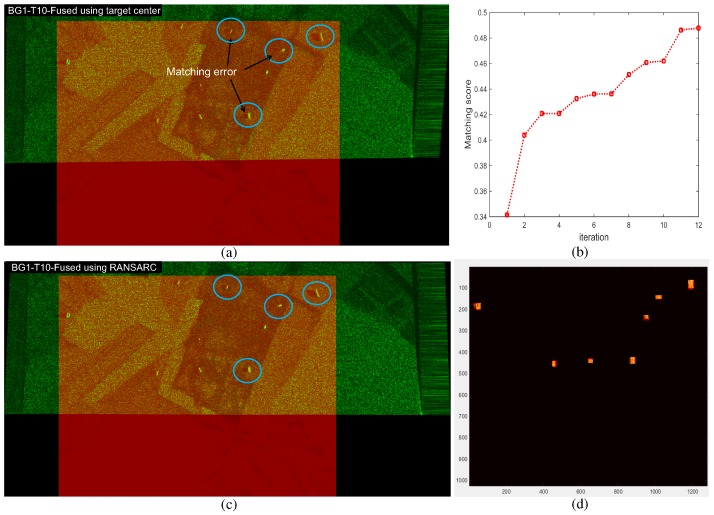
SAR and IR registration results for the background ID1 using BG1-T10-IR/SAR images: (**a**) initial automatic registration using a brute-force search from the detected target centers; (**b**) registration optimization curve of the proposed RANSARC; (**c**) refined registration results; (**d**) detected region overlap by the estimated homography.

**Figure 25:**
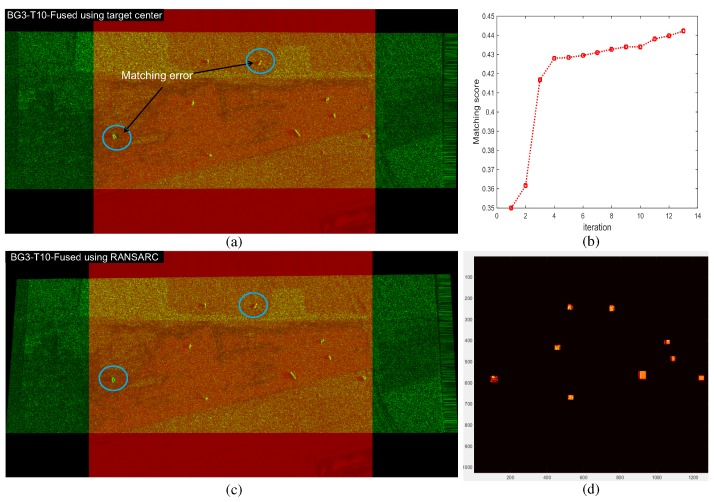
SAR and IR registration results for the background ID3 using BG3-T10-IR/SAR images: (**a**) initial automatic registration using a brute-force search from the detected target centers; (**b**) registration optimization curve of the proposed RANSARC; (**c**) refined registration results; (**d**) detected region overlap by the estimated homography.

SAR and IR image registration is required to detect the final targets. Because SAR and IR images have completely different characteristics, it is difficult to find the corresponding points to estimate the image homography. This paper proposes a RANSARC method to register heterogeneous SAR and IR images using the target points and target regions. The first step is to find the initial homography by a brute-force correspondence search from the detected target centers. The second step is to optimize the homography by applying RANSARC to the detected regions. RANSARC can maximize the overlapping ratio between the SAR and IR regions. The proposed method is then applied to backgrounds 1, 2 and 3 to register the SAR and IR images. The registration information is used in the following sensor fusion-based target detection. The registration results of background 2 is explained in Figure 16. Figure 24 and Figure 25 present the remaining registration results of backgrounds 1 and 3, respectively. Test SAR/IR images containing maximum number of targets (BG1-T10-SAR/IR, BG3-T10-SAR/IR) were used to increase the accuracy of image registration. If only the target center points are used, there would be large registration errors (approximately 10–15 pixels in distance), as shown in Figure 24a and Figure 25a. Through the RANSARC-based optimization process (see Figure 24b and Figure 25b), the SAR and IR images were registered with reduced error (under 5 pixels), as shown in Figure 24c and Figure 25c. Figure 24d and Figure 25d show the finally overlapped target regions by the optimal image homography.

### 4.4. SAR/IR Fusion Based Final Target Detection

**Figure 26:**
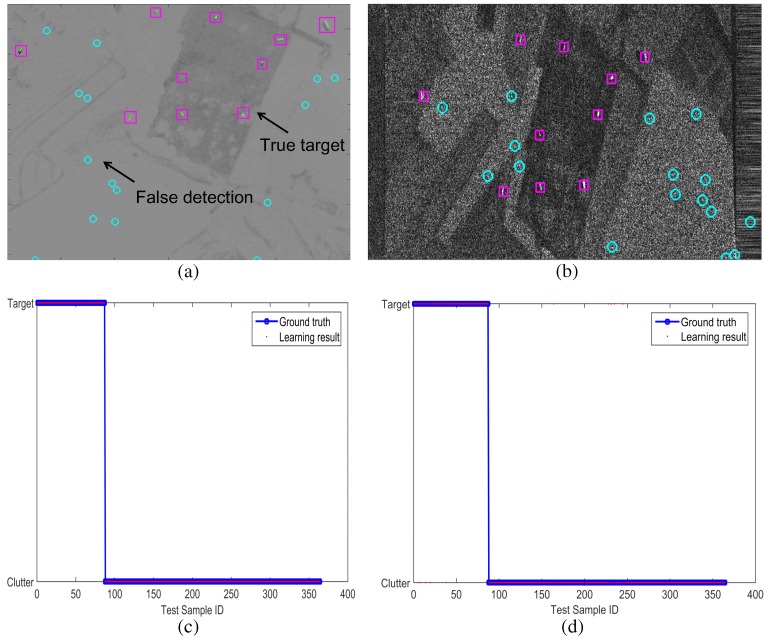
Preparation of training data and trained results: (**a**) true targets and false detections in an IR image (BG1-T10-IR); (**b**) true targets and false detection is an SAR image (BG1-T10-SAR); (**c**) trained results of Adaboost (error rate 0%); (**d**) trained results of LapSVM (error rate 3%).

Until now, the basic components of independent target detection and the registration method were evaluated. In this subsection, the performance of the final target detection schemes was evaluated in the terms of fusion schemes, such as `without sensor fusion’ and `with sensor fusion’ in noisy environment. In `without sensor fusion’ scheme, the modBMVT-based SAR and IR detection method are used. In `with sensor fusion’, learning-based fusion methods, such as Adaboost, Laplacian SVM (LapSVM) method are compared. Adaboost is used frequently in face detection or car detection [52]. In this study, the method is used as a feature selection-based SAR/IR fusion scheme. LapSVM is selected as a baseline feature-level fusion to compare it with Adaboost because LapSVM is the state-of-the-art classifier in semi-supervised learning [49]. In this paper, the open source code in the web page [53] was used for a fair comparison. In addition, another decision-level logical fusion, such as AND/OR are also considered. Training features were extracted from the ground truth and false detections, as shown in Figure 26a,b. The signal in the enhanced map (modBMVT) and target area were used as features in both SAR and IR images. Gaussian noise was inserted in the IR images and the detection threshold in the modBMVT was set to a low value to verify the effects of the fusion scheme. The BG1-T10-IR/SAR, BG2-T10-IR/SAR, and BG3-T10-IR/SAR images were used for training and the remaining images were used for testing the target detection. Figure 26c,d shows the training results of Adaboost and LapSVM using 90 true targets and 270 false targets. Adaboost was trained perfectly and LapSVM produced a 3.0% error for the training data. The error was caused by the learning structure of LapSVM, which is a semi-supervised learning [49].

Table 1 lists the overall target detection performance for the different fusion schemes. As performance metrics, the detection rate and false alarms per image were used for this purpose. IR only (without fusion) produced a relatively low detection rate with a high rate of false alarms. SAR only produced a relatively high detection rate (96.9%) with high false alarms. The proposed feature-selection-based fusion method (Adaboost) showed the best detection rate (100%) with the lowest number of false alarms (4.1 false alarms/image). The next best was LapSVM-based fusion method. Note that Adaboost and LapSVM used a default decision making method (no bias is controlled). The simple logical AND fusion method showed the lowest detection rate with a relatively low number of false alarms. Although the logical OR method produced a perfect detection rate, it showed the highest number of false alarms.

**Figure 27:**
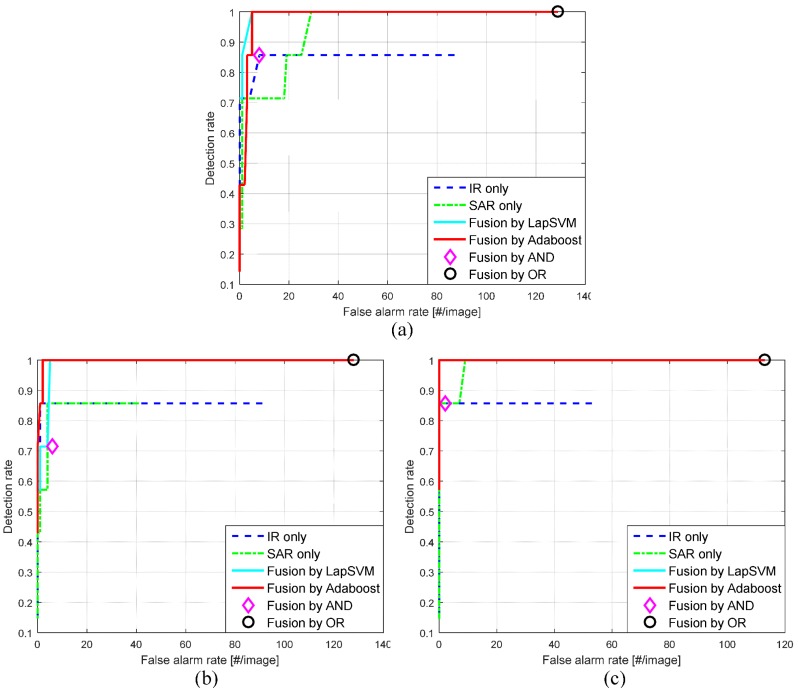
Comparison by ROC curves: (**a**) result of a test image, BG1-T7-IR/SAR; (**b**) result of an test image, BG2-T7-IR/SAR; (**c**) result of an test image, BG3-T7-IR/SAR.

**Figure 28:**
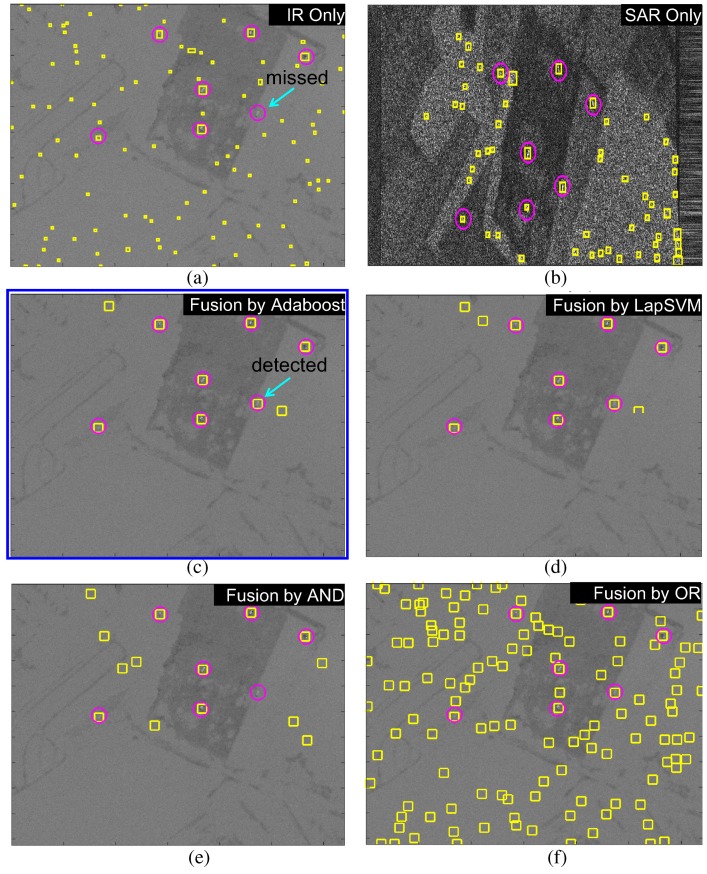
Comparison of target detection on the BG1-T7-IR/SAR DB: (**a**) without fusion (IR only); (**b**) without fusion (SAR only); (**c**) proposed Adaboost-based fusion; (**d**) LapSVM-based fusion; (**e**) logical AND fusion; and (**f**) logical OR fusion.

**Figure 29:**
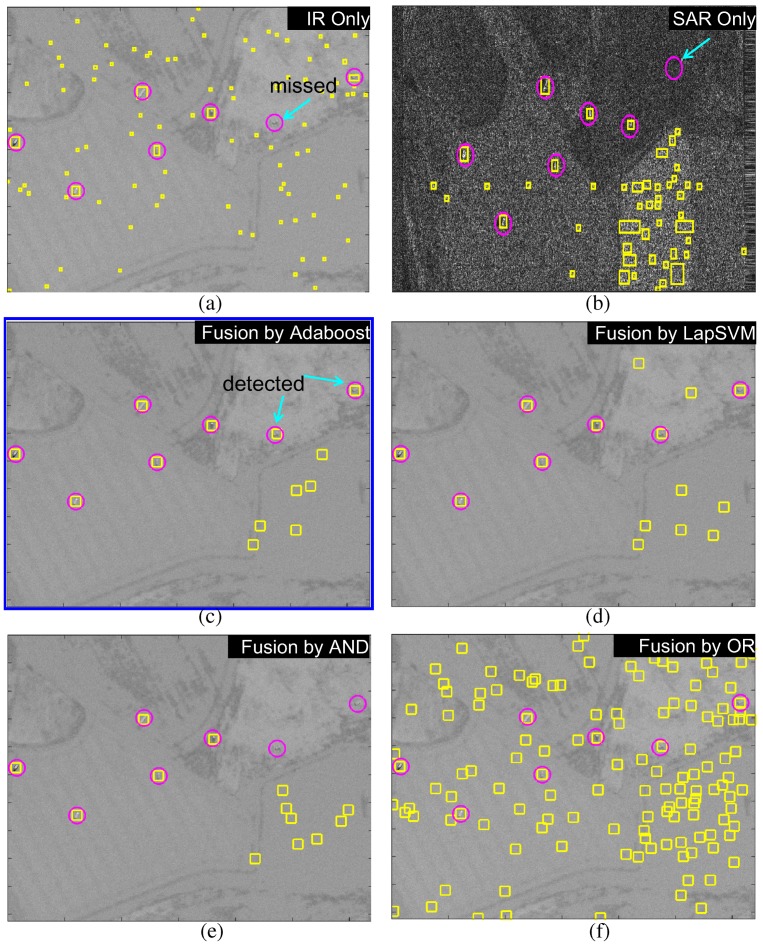
Comparison of target detection on the BG2-T7-IR/SAR DB: (**a**) without fusion (IR only); (**b**) without fusion (SAR only); (**c**) proposed Adaboost-based fusion; (**d**) LapSVM-based fusion; (**e**) logical AND fusion; and (**f**) logical OR fusion.

In addition, the target detection accuracies were measured by the receiver operating characteristic (ROC) curves, as shown in Figure 27. Three types of test images were evaluated by controlling the thresholds in the detectors except AND or OR fusion, which does not have any control parameters. According to the overall comparison, the Adaboost-based fusion method showed the best performance followed by the LapSVM-based fusion method. Figure 28 and Figure 29 present the comparative results using different fusion schemes for the test images (BG1-T7-IR/SAR, BG2-T7-IR/SAR). The circles denote the ground truth target locations and the rectangles denote detected target regions. Note that missed targets in the IR or SAR sensor were detected correctly in the Adaboost-based fusion method. The LapSVM-based fusion method showed comparable detection performance with Adaboost.

## 5. Conclusions and Discussion

This paper proposed a novel SAR/IR target detection by feature selection-based fusion from candidate targets generated using a modified Boolean map visual theory-based method (modBMVT). This paper attempted to solve several issues encountered in SAR/IR fusion-based target detection research. The most fundamental problem was acquiring the SAR/IR target images. No open database is available due to security reasons. This paper proposes the use of the SAR/IR image generation tool, OKTAL-SE, which can produce SAR and IR images for the same scenario. Although the synthesized images are not perfect, they are useful in the beginning of fusion research. The second issue is making a suitable SAR/IR target detector that can be effective in both the SAR and IR domain. There can be the best detector for each sensor domain. On the other hand, developing sensor-dependent detectors can be time-consuming and expensive in implementation. In this paper, a modified Boolean map visual theory-based SAR/IR detection method was proposed by attaching a pre-filter and post-filter to the conventional BMVT. The pre-filter, called the median local average filter (MLAF), can effectively remove both thermal (in IR image) and scattering noise (in SAR image). The post-filter, called the asymmetric morphological closing filter (AMCF), can reduce the partitioned detection points. The third issue was registering the SAR/IR images automatically. The corresponding points between SAR and IR are very difficult to find because they image completely different physical characteristics. This paper proposed a two step-based registration method. The initial homography was estimated by a brute-force correspondence search from the detected target centers assuming sufficient target numbers (>4). The final homography was optimized by RANdom SAmple Region Consensus (RANSARC). The proposed RANSARC can refine the initial homography by optimally aligning the detected regions. The final problem is how to fuse the heterogeneous sensor data. This paper proposed a feature selection-based sensor fusion using Adaboost. Although this study used simple filtered signal and area, the Adaboost-based sensor fusion showed the best detection performance in terms of the detection rate and false alarm rate compared to the other fusion methods, such as LapSVM and Logical AND/OR. Future studies will consider different target features, such as ranked-fill-ratio, 2nd-order moment, size ratio, rotational size variation, and frequency energy to improve the fusion performance. In addition, the proposed method should be applied to real SAR and IR images, which is the most challenging area.

## Figures and Tables

**Table 1:** Comparison of the target detection performance.

Fusion Scheme	Detection Rate (%)	False Alarms/Image
IR only	84.8 (28/33)	74.6 (448/6)
SAR only	96.9 (32/33)	50.1 (301/6)
**Proposed (Adaboost)**	**100.0 (33/33)**	**4.1 (25/6)**
LapSVM [49]	100.0 (33/33)	6.1 (37/6)
Logical AND [46]	81.8 (27/33)	4.8 (29/6)
Logical OR [46]	100.0 (33/33)	119.3 (716/6)
